# Supplementary material for: A primordial noble gas component discovered in the Ryugu asteroid and its implications
Source: Nat Commun. 2024 Sep 14;15:8075. doi: 10.1038/s41467-024-52165-0 (PMC11401872; doi:10.1038/s41467-024-52165-0)
Supplement: Supplementary file 1 — Supplementary Information [file 41467_2024_52165_MOESM1_ESM.pdf]

Supplementary materials

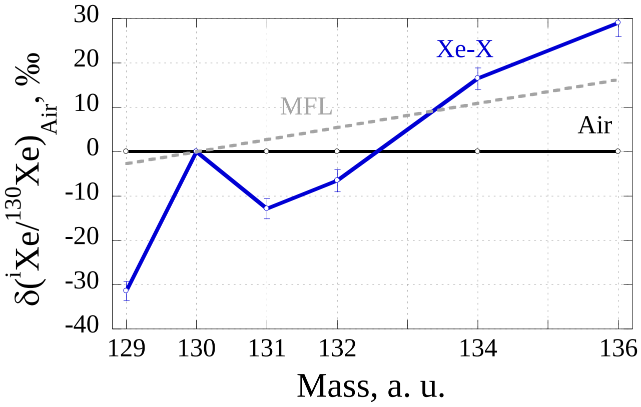

Figure S1. Deviations of Xe-X isotope ratios from air Xe when normalised to ^{130}Xe. It is clear that Xe-X cannot be produced from air Xe by simple mass-dependent fractionation. Error bars correspond to 1σ. Source data are provided in the Table S3.

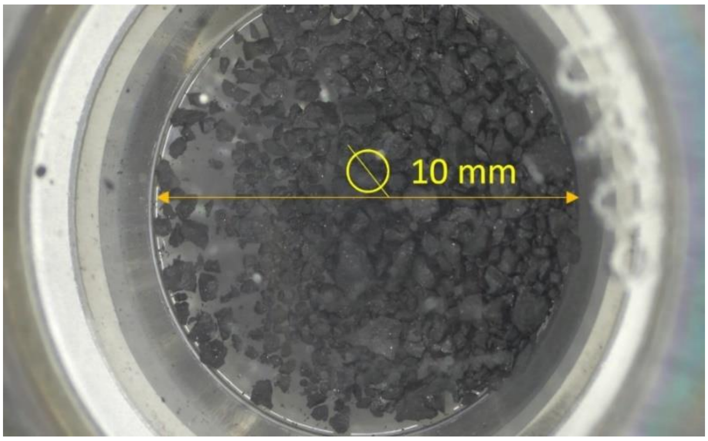

Figure S2. Picture of sample A0219. The other analysed Ryugu samples look similar.

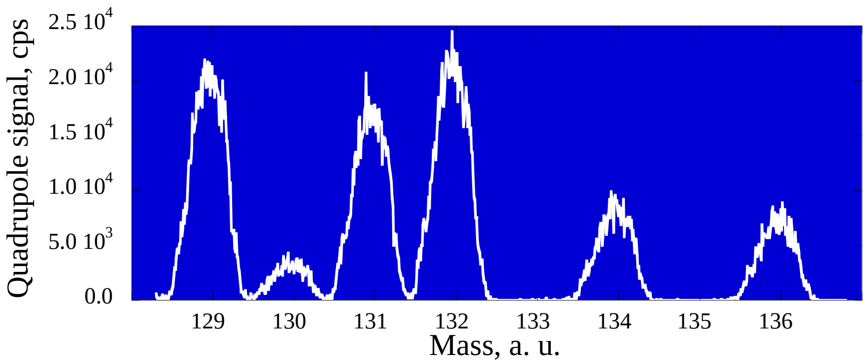

Figure S3. Mass spectrum of air Xe. The amount of Xe is about 7×10^{-11} cm^3 STP. Source data are provided as a Source Data file with corresponding sheet name.

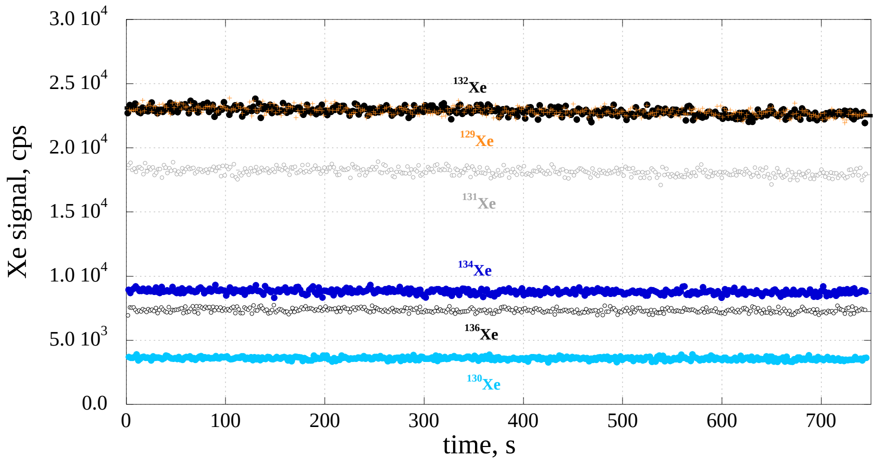

Figure S4. Xe peak intensity variations depending on time during analysis of ~7x10^{-11} cc STP of Xe air standard on the quadrupole mass spectrometer. Source data are provided as a Source Data file with corresponding sheet name.

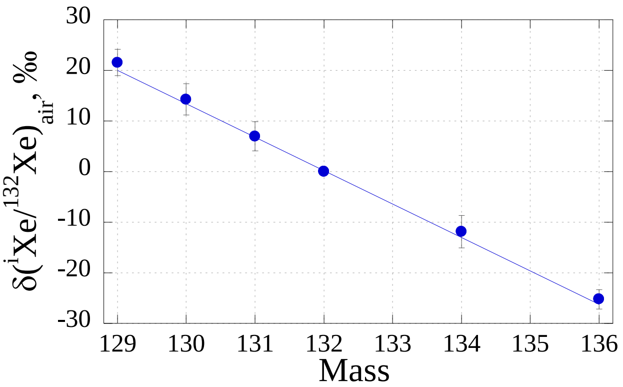

Figure S5. Mass discrimination of standard air Xe in the quadrupole mass spectrometer. Error bars – 1σ. Source data are provided as a Source Data file with corresponding sheet name.

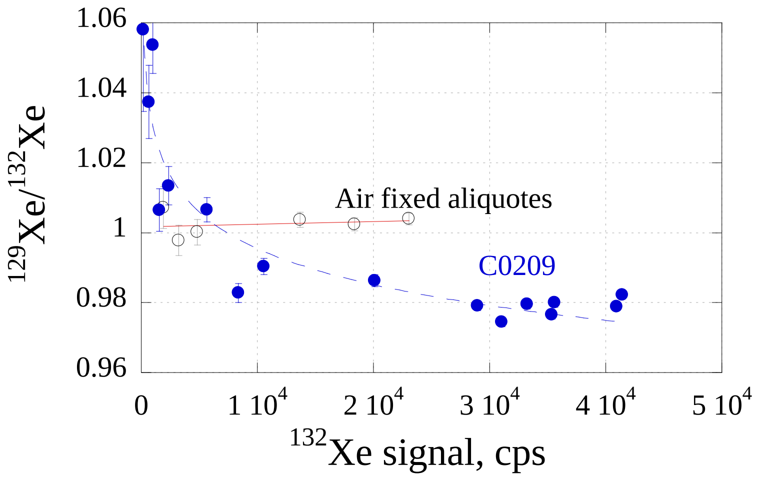

Figure S6. ^{129}Xe/^{132}Xe ratio variations depending on the amount of Xe in the quadrupole mass spectrometer for air reference (open cercles) and for temperature steps of the sample C0209 (filled cercles). Since air Xe does not show significant variations in the isotope ration on the amount of Xe,

the variations for the sample are due to mixing of isotopically different components. Solid (back) and blue (dotted) lines are best fits for the corresponding experimental points. Error bars correspond to 1σ. Source data are provided as a Source Data file with corresponding sheet name.

Table S1. ^{4}He, ^{20}Ne and ^{36}Ar concentrations and Ne isotopic compositions in the temperature (combustion) steps of the C0209 Ryugu sample.

T, ºC	^{4}He, cc STP/g x10^{-5}	^{20}Ne, cc STP/g x10^{-7}	$^{20}Ne/^{22}Ne$	$^{21}Ne/^{22}Ne$	^{36}Ar, cc STP/g x10^{-8}
C0209, 2.46 mg					
100	0.12	n.a	n.a	n.a	0.27
150	5.41	3.21	12.53(64)	0.0248(45)	1.69
200	7.62	4.65	13.31(55)	0.0396(35)	3.25
225	5.64	3.71	12.18(57)	0.0368(40)	3.47
250	7.19	5.63	12.80(44)	0.0396(31)	5.85
275	7.66	7.18	12.62(36)	0.0465(26)	9.42
300	7.12	7.08	13.02(40)	0.0470(31)	12.0
325	8.54	8.16	13.13(37)	0.0362(26)	14.3
350	7.96	8.41	12.53(34)	0.0375(24)	15.6
375	6.14	8.13	12.82(39)	0.0424(24)	16.2
400	4.57	7.30	12.67(43)	0.0302(24)	16.1
425	3.66	7.27	12.67(46)	0.0420(27)	18.4
450	2.89	6.99	12.98(53)	0.0401(30)	20.5
475	2.32	6.22	12.74(59)	0.0338(30)	21.1
500	0.71	9.27	12.05(37)	0.0386(19)	18.3
525	0.63	7.77	11.71(44)	0.0323(22)	12.6
550	0.42	1.78	11.5(19)	0.0453(97)	4.86
575	b.l.	1.57	13.0(30)	0.053(14)	4.39
600	b.l.	1.99	13.4(27)	0.0371(88)	4.03
625	b.l.	b.l.	n.a	n.a	2.69
650	b.l.	b.l.	n.a	n.a	2.94
700	0.34	b.l.	n.a	n.a	6.73
750	0.70	b.l.	n.a	n.a	1.36
800	0.83	0.17	n.a	n.a	1.02
900	1.51	1.27	n.a	n.a	2.48
1000	0.92	1.12	n.a	n.a	0.57
1100	0.03	b.l.	n.a	n.a	1.13
1200	b.l.	b.l.	n.a	n.a	0.42
1400	b.l.	b.l.	n.a	n.a	0.28
total	82.9	109	12.64(54)	0.0386(31)	243.0

Errors (1σ) for Ne isotopic composition are shown in parentheses and related to the last two digits. b.l.- amounts of gas do not exceed blank level. n.a. – not analysed.

Table S2. ^{4}He, ^{20}Ne and ^{36}Ar concentrations and Ne isotopic compositions in the temperature (combustion) steps of the C0208 Ryugu sample.

T, ºC	^{4}He, cc STP/g x10^{-5}	^{20}Ne, cc STP/g x10^{-7}	$^{20}Ne/^{22}Ne$	$^{21}Ne/^{22}Ne$	^{36}Ar, cc STP/g x10^{-8}
C0208, 1.7 mg					
100	b.l.	0.05	n.a	n.a	1.10
150	9.79	3.93	12.88(61)	0.0269(50)	2.71
200	12.5	6.27	12.75(39)	0.0235(39)	2.98
225	10.9	7.10	13.20(39)	0.0287(36)	6.11
250	14.3	11.8	13.35(27)	0.0381(25)	9.52
275	13.6	15.6	12.92(20)	0.0399(22)	12.1
300	13.5	17.4	13.38(20)	0.0327(21)	1.68
325	16.2	20.9	13.18(18)	0.0307(18)	17.0
350	14.9	20.1	13.23(19)	0.0319(18)	18.5
375	12.0	18.8	13.33(22)	0.0326(20)	20.0
400	9.66	18.1	13.32(24)	0.0359(23)	20.3
425	6.99	15.7	13.38(28)	0.0319(25)	22.2
450	5.47	14.9	13.37(30)	0.0317(26)	22.6
475	4.17	13.3	13.40(35)	0.0342(26)	20.2
500	3.32	11.4	13.66(45)	0.0360(33)	14.8
525	2.36	8.98	13.76(60)	0.0383(43)	11.1
550	0.98	6.62	14.62(95)	0.0368(58)	9.04
575	0.57	4.81	n.a	n.a	6.87
600	0.45	2.88	n.a	n.a	12.6
650	0.87	3.01	n.a	n.a	21.6
700	1.22	0.84	n.a	n.a	43.6
750	1.49	b.l.	n.a	n.a	33.9
800	1.50	b.l.	n.a	n.a	3.43
850	1.29	b.l.	n.a	n.a	3.14
900	0.87	b.l.	n.a	n.a	3.12
1000	1.14	1.55	n.a	n.a	1.81
1100	0.03	b.l.	n.a	n.a	1.14
1200	b.l.	b.l.	n.a	n.a	0.05
1400	b.l.	0.12	n.a	n.a	b.l.
total	160.0	224.0	13.33(30)	0.0336(26)	359.0

Errors (1σ) for Ne isotopic composition are shown in parentheses and related to the last two digits. b.l.- amounts of gas do not exceed blank level. n.a. – not analysed.

Table S3. ^{4}He, ^{20}Ne and ^{36}Ar concentrations and Ne isotopic compositions in the temperature (combustion) steps of the A0219 Ryugu sample.

T, °C	^{4}He, cc STP/g x10^{-5}	^{20}Ne, cc STP/g x10^{-7}	$^{20}Ne/^{22}Ne$	$^{21}Ne/^{22}Ne$	^{36}Ar, cc STP/g x10^{-8}
A0219, 2 mg					
100	2.83	b.l.	n.a	n.a	0.52
150	2.05	0.82	n.a	n.a	1.23
200	2.98	2.01	n.a	n.a	3.39
250	5.02	6.07	13.71(50)	0.044(16)	8.75
300	6.28	14.8	12.86(21)	0.0401(50)	22.7
325	3.57	8.10	13.50(40)	0.0319(70)	16.1
350	3.47	10.3	16.08(44)	0.0331(72)	14.3
375	3.30	10.5	13.39(33)	0.0352(61)	18.7
400	2.79	11.7	12.74(26)	0.0274(47)	23.3
425	1.96	11.9	12.77(27)	0.0272(46)	24.2
450	1.61	12.4	13.33(30)	0.0376(49)	28.4
475	1.28	11.2	13.66(33)	0.0298(53)	24.4
500	1.62	10.9	13.36(32)	0.0323(53)	21.1
525	1.32	9.90	13.30(33)	0.0418(73)	13.0
550	0.29	8.44	13.90(39)	0.0263(50)	9.34
575	0.05	6.64	13.66(48)	0.048(11)	7.32
600	b.l.	4.94	13.02(50)	0.035(11)	5.53
650	0.12	4.63	12.26(51)	0.040(14)	10.8
700	0.30	2.26	11.70(87)	0.059(44)	21.3
800	0.84	1.62	n.a	n.a	69.5
900	0.61	2.24	n.a	n.a	6.91
1000	0.21	2.36	n.a	n.a	2.19
1200	b.l.	3.85	n.a	n.a	2.35
1400	b.l.	b.l.	n.a	n.a	0.03
total	42.5	157.0	13.38(35)	0.0350(73)	356.0

Errors (1σ) for Ne isotopic composition are shown in parentheses and related to the last two digits. b.l.- amounts of gas do not exceed blank level. n.a. – not analysed.

Table S4. Xe concentration and isotopic composition in the temperature (combustion) steps of C0209 and C0208 Ryugu samples.

T, °C	^{132}Xe, cc STP/g x10^{-8}	^{136}Xe/^{132}Xe	^{134}Xe/^{132}Xe	^{131}Xe/^{132}Xe	^{130}Xe/^{132}Xe	^{129}Xe/^{132}Xe
C0209, 2.46 mg						
100	0.04	0.324(10)	0.3486(96)	0.689(28)	0.1513(72)	0.897(18)
150	1.13	0.3376(17)	0.3912(19)	0.7802(34)	0.15191(95)	0.9621(36)
200	4.18	0.3415(12)	0.3950(15)	0.7832(25)	0.15236(64)	0.9539(28)
225	3.89	0.3421(12)	0.3961(15)	0.7844(26)	0.15153(64)	0.9585(28)
250	4.79	0.3438(12)	0.3971(15)	0.7837(26)	0.15154(61)	0.9594(27)
275	5.51	0.3405(11)	0.3966(14)	0.7857(25)	0.15306(60)	0.9582(27)
300	5.57	0.3432(11)	0.3970(14)	0.7866(25)	0.15348(60)	0.9615(26)
325	4.76	0.3401(11)	0.3978(15)	0.7851(25)	0.15305(62)	0.9560(27)
350	4.47	0.3412(12)	0.3961(15)	0.7833(26)	0.15199(62)	0.9589(27)
375	2.71	0.3410(13)	0.3935(16)	0.7880(27)	0.15255(73)	0.9655(29)
400	1.42	0.3384(15)	0.3910(18)	0.7907(31)	0.15212(86)	0.9695(33)
425	0.76	0.3366(19)	0.3951(22)	0.7901(39)	0.1543(12)	0.9855(42)
450	0.32	0.3287(27)	0.3887(30)	0.7975(58)	0.1573(17)	0.9924(60)
475	0.21	0.3309(33)	0.3993(35)	0.8125(73)	0.1569(21)	0.9857(66)
500	0.13	0.3472(41)	0.4010(47)	0.824(10)	0.1627(30)	1.033(87)
525	0.09	0.3535(51)	0.4083(58)	0.824(14)	0.1680(37)	1.02(11)
550	0.02	0.379(14)	0.415(13)	0.818(44)	n.a	1.04(27)
total	40.0	0.3412(13)	0.3960(16)	0.7855(28)	0.15260(69)	0.9604(29)
C0208, 1.7 mg						
100	0.021	*0.294*	0.379(22)	n.a.	n.a.	*1.026*
150	0.049	0.319(12)	0.389(11)	*0.682*	*0.129*	*0.958*
200	0.039	0.328(14)	0.394(14)	*0.897*	0.142(11)	1.029(28)
225	0.048	0.340(12)	0.385(11)	*0.734*	0.1444(93)	1.045(23)
250	0.093	0.317(10)	0.3823(68)	0.802(19)	0.1450(52)	1.036(14)
275	0.147	0.3069(51)	0.3691(51)	0.802(13)	0.1495(37)	1.030(11)
300	0.169	0.3024(45)	0.3641(47)	0.803(12)	0.1495(33)	1.037(10)
325	0.176	0.3087(45)	0.3860(45)	0.830(11)	0.1440(33)	1.037(10)
350	0.167	0.3295(46)	0.3810(48)	0.836(12)	0.1665(45)	*1.073*
375	0.168	0.3074(47)	0.3737(48)	0.809(12)	0.1575(33)	*0.988*
400	0.159	0.3232(48)	0.3817(49)	0.816(12)	0.1531(36)	1.021(10)
425	0.134	0.3094(53)	0.3873(55)	0.802(12)	0.1611(40)	1.056(12)
450	0.111	0.3195(50)	0.3849(61)	0.790(14)	0.1665(45)	1.005(12)
475	0.097	0.3333(69)	0.3968(68)	0.873(19)	*0.180*	1.029(14)
500	0.078	0.3553(82)	0.3890(78)	0.844(22)	*0.165*	1.013(17)
525	0.047	0.378(12)	0.441(11)	0.827(35)	*0.141*	1.012(22)
550	0.027	0.342(19)	0.385(17)	*0.684*	*0.159*	1.004(35)
575	0.012	*0.332*	0.367(29)	n.a.	n.a.	*1.007*
total	1.78	0.3200(65)	0.3742(64)	0.815(14)	0.1540(41)	1.033(13)

Errors (1σ) for Xe isotopic composition are shown in parentheses and related to the last two digits. Numbers in italics are not reliable measurements and were not used for calculations of the totals. Above 550°C for C0209 and 575°C for C0208 only 0.1% and 2% of Xe are released, respectively. n.a. – not analysed. The highlighted Xe isotope ratios for C0209 and ^{129}Xe/^{132}Xe ratios for C0208 were used to plot Fig. 3 and calculate Xe-X average isotopic composition shown in Figure 3 in the main text and Table S5.

Table S5. Xe-X (P7) isotope ratios with two alternative normalizations.

**$^{136}Xe/^{132}Xe$**	**$^{134}Xe/^{132}Xe$**	**$^{131}Xe/^{132}Xe$**	**$^{130}Xe/^{132}Xe$**	**$^{129}Xe/^{132}Xe$**
0.3412±0.0006	0.3969±0.00030	0.7840±0.0007	0.15236±0.00021	0.9586±0.0009
**$^{136}Xe/^{130}Xe$**	**$^{134}Xe/^{130}Xe$**	**$^{132}Xe/^{130}Xe$**	**$^{131}Xe/^{130}Xe$**	**$^{129}Xe/^{130}Xe$**
2.239±0.005	2.605±0.004	6.563±0.016	5.146±0.009	6.292±0.010

Errors correspond to 1σ.
